# Health Monitoring and Evaluation of Long-Span Bridges Based on Sensing and Data Analysis: A Survey

**DOI:** 10.3390/s17030603

**Published:** 2017-03-16

**Authors:** Jianting Zhou, Xiaogang Li, Runchuan Xia, Jun Yang, Hong Zhang

**Affiliations:** College of Civil Engineering, Chongqing Jiaotong University, Chongqing 400074, China; li.xiaogang@tylin.com.cn (X.L.); 622150086089@mails.cqjtu.edu.cn (R.X.); 611150086003@mails.cqjtu.edu.cn (J.Y.); hongzhang@cqjtu.edu.cn (H.Z.)

**Keywords:** sensor, bridge health monitoring, safety evaluation

## Abstract

Aimed at the health monitoring and evaluation of bridges based on sensing technology, the monitoring contents of different structural types of long-span bridges were defined. Then, the definition, classification, selection principle, and installation requirements of the sensors were summarized. The concept was proposed that new adaptable long-life sensors could be developed by new theories and new effects. The principle and methods to select controlled sections and optimize the layout design of measuring points were illustrated. The functional requirements were elaborated on about the acquisition, transmission, processing, and management of sensing information. Some advanced concepts about the method of bridge safety evaluation were demonstrated and technology bottlenecks in the current safety evaluation were also put forward. Ultimately, combined with engineering practices, an application was carried out. The results showed that new, intelligent, and reliable sensor technology would be one of the main future development directions in the long-span bridge health monitoring and evaluation field. Also, it was imperative to optimize the design of the health monitoring system and realize its standardization. Moreover, it is a heavy responsibility to explore new thoughts and new concepts regarding practical bridge safety and evaluation technology.

## 1. Introduction

As lifeline engineering, bridges are in a stage of rapid development at present, and the community has attached great importance to the operational safety of bridges. Therefore, how to ensure the healthy operation of long-span bridges has become a hot topic that is difficult to address in this field. Through the service period that ranges from decades to even a hundred years, bridges will be affected by external loads like cars, climate, and rivers. Bridges will also be affected by internal effects like material aging, fatigue, and other factors [1]. These factors will result in inevitable damage and serious accidents. For example, on 1 August 2007, the I-35W Mississippi River Bridge in the United States collapsed [2]. On 14 July 2011, the Wuyishan Mansion Bridge in the Fujian province of China collapsed [3]. The lessons taught by a series of disasters have made health monitoring and evaluation for bridges a major issue.

Sensing technology belongs to modern science and its purpose is to obtain and identify the information from natural sources by sensors. With the innovation of technology, sensors that are original, intelligent, and reliable have been widely used for health monitoring of long-span bridges. For instance, sensors were installed on the Foyle Bridge [4], which is a continuous steel bridge with variable heights and a total length of 522 m. Their purpose was to monitor the response of vibration, deflection, and strain under the vehicle and wind load in the operation stage. Numerous acceleration sensors and strain gauges were attached to the Tsing Ma Bridge with a set of GPS (Global Position System) devices, which were used for the long-term monitoring of the service safety [5]. Monitoring systems have also been installed in numerous bridges. For example, in Japan, the Akashi Kaikyo Bridge [6] has a main span of 1991 m. In Denmark, there exists the Great Belt East Suspension Bridge and the Faroe Sea-Crossing Cable-Stayed Bridge, of which the main spans are 1624 m and 1726 m, respectively. In Norway, the Skarnsundet Cable-Stayed Bridge has a main span of 530 m. Additionally in China, there exists the Su Tong Yangtze River Highway Bridge and the Run Yang Yangtze River Bridge, of which the main spans are 1088 m and 1490 m, respectively. In this paper, the Caijia Jialing River Bridge for the subway in Chongqing city, China, will be taken as an example. Its total length is 1250 m. Also, constant, on-line, and dynamic health monitoring is conducted so as to achieve the objective of real and effective evaluation of its structural state [7,8,9].

With the progress and development of technology, the current health monitoring program focuses on smart sensors and methods of reliability and safety evaluation. The implementation relies on the monitoring system [10]. A set of complete monitoring systems has functions such as acquisition, transmission, processing, management, evaluation, early warning, etc. The whole process is completed by six systems [11].

(1) The sensor system is aimed at obtaining load, environment, and structural response information.
(2) The data acquisition and transmission system is aimed at signal acquisition, caching, and transmission.
(3) The data processing and control system is aimed at routine data processing and analysis, system status monitoring, remote control, and structural warning.
(4) The evaluation system is aimed at executing the original monitoring data analysis, structural status evaluation, diagnosis, and prediction.
(5) The data management system is aimed at storage and extraction of the monitoring data and analysis results.
(6) The inspection and maintenance system is aimed at sensors for inspection and maintenance, data acquisition unit, data transmission network and display equipment, etc.

With respect to bridge safety evaluation technology, the applied theories mainly include the reliability theory, analytic hierarchy process, fuzzy theory, etc.

In summary, the health monitoring of long-span bridges has achieved great progress. To obtain standardized management, many countries have promulgated relevant guidelines and procedures. For example, ISIS (Intelligent Sensing for Innovative Structures) in Canada released “The Structural Health Monitoring Guide” [12]. Drexel University in America completed “A Case Study on the Health Monitoring Guide for Significant Bridges” [13] with the FHWA (Federal Highway Administration). SAMCO (Structural Assessment, Monitoring and Control Organization) released “The Structural Health Monitoring Guide” [14] with the European Union. In China, the department of transportation released “Technical specification for structural safety monitoring systems of highway bridges” [15]. However, when they were applied to engineering practices, there were still some obvious shortcomings, such as the mechanism and technology of monitoring and sensing, monitoring theory and technology of the service lifetime, the processing of monitoring information [16], structural status evaluation, etc. Also, the combined effects of the complex service environment and disaster loads can accelerate the damage evolution of long-span bridges, which will have negative results on the health and safety of the overall structure. This should be considered with the planning and construction of super bridge projects across long-span seas or rivers, such as the Shanghai Railway Yangtze River Bridge, the Egongyan Special Track Bridge in China, the Pulau Muara Besar Bridge, etc. The monitoring and evaluation tasks will become more burdensome and difficult. As a result of the health monitoring and evaluation of long-span bridges based on sensing technology, the research status, positive innovation, and the scientific grasp of damage evolution laws need to be recognized to ensure the safety of the structures [17].

## 2. Health Monitoring Contents

Via physical and mechanical properties, the health monitoring of long-span bridges is aimed at monitoring the overall structures non-destructively and constantly. Then, the location and degree of structural damages can be diagnosed. Also, the service situation, reliability, durability, and bearing capacity can also be evaluated. If the structure has an emergency or severely abnormal usage, warning signals will be triggered to provide a basis and guidance for maintenance, management, and decision-making [18]. Due to different types of long-span bridges combined with differences in the operating environment as well as the need for monitoring functions, the monitoring contents should be chosen appropriately, which are shown in Table 1.

## 3. Sensing Technology

### 3.1. Sensor

The sensor is a device that can detect certain specified parameters and convert them into available signals. According to the principle of operation, it can be classified by resistance, raster, piezoelectric, laser [19], etc. As the leading edge of health monitoring systems, the selection of the sensor should follow the principle of “stable and reliable performance which is cost-effective” and should be convenient for the integration of the health monitoring system. Also, some evaluation indexes, such as sensitivity, linearity, signal-to-noise ratio, and resolution should also meet the monitoring needs. Before the installation of the sensor, it is necessary to carry out its calibration. During the installation process, damage to the structure should be reduced.

With the rapid development of science and technology, there has been some breakthroughs in the long-term stability, reliability, durability, and other aspects of sensors. Meanwhile, some new types of practical sensors have emerged, such as the multi-directional dynamic stress monitoring sensor for concrete [20]. However, the information acquisition of bridge health monitoring and evaluation are greatly restricted because of the complexity of the working environment, the mismatch between the long life of the bridge and the short life of the sensors, and the monitoring signals which result from multi-effects. Therefore, by using new theories and new effects, in the future it will be a hot topic to study and develop new types of long-life sensors with stronger adaptability.

### 3.2. Optimum Layout of the Sensors

Due to the limited number of measuring points in health monitoring systems, the full freedom data of long-span bridges cannot be obtained. Therefore, it is necessary to optimize the sensor configuration so as to achieve effective damage identification.

Considering the comprehensiveness, accuracy, and economic factors regarding constant response information, when sensors are arranged, the controlled sections should be selected by following multiple principles of analyzing internal forces and deformation, vulnerability, and geometric dimensions. Methods include technology of static control analysis, dynamic control analysis, and monitoring overall bridge state acquisition via finite points.

According to the criterion of minimum errors of identification (transmission), model reduction [21], interpolation fitting, and MAC (Modal Assurance Criterion) [22], some methods can be used to optimize the layout of sensors. The methods include genetic algorithms, data fusion, inversion theory, and optimization which is combined with construction monitoring.

## 4. Sensing Information

### 4.1. Data Acquisition

According to the structure type of long-span bridges, the number of health monitoring points, the type of sensor, the acquisition mode of sensing information, etc., should be rationally designed:

(1) If the measuring points are relatively far apart and scattered, the distributed acquisition mode should be adopted.
(2) If the measuring points are relatively close to each other and concentrated, the centralized acquisition mode or that combined with the distributed mode should be adopted.

The acquisition device can be divided into hardware and software. The hardware device should follow the standard protocol and interface with the function of constant acquisition, automatic storage, and instant display. The software device can achieve manual intervention acquisition and adjust parameters, preliminarily dealing with the output contents of sensing information and the identification of abnormal information.

Sensor devices with special software to measure and control should be provided with the software running platform, communication protocol, and response interface. As for the acquisition software of the development platform, the language should meet the requirements of health monitoring visualization where LabVIEW [23] and LabWindows [24] can be adopted.

### 4.2. Data Transmission

Combined with the transmission distance, network coverage, communication facilities, and other factors, reasonable transmission modes of sensing information are respectively selected, which are then followed.

(1) If the monitoring range is small, it shall be based on the signal synchronization technology.
(2) If the monitoring range is large, it shall be based on the time synchronization technology [25].
(3) If the electromagnetic interference cannot be shielded in bridge monitoring, cable transmission shall be adopted.
(4) If it is in surroundings where the layout and the maintenance of wires are difficult, or if it is necessary to build the monitoring structure which can temporarily transmit network data, wireless transmission [26] shall be adopted.
(5) According to the needs of the projects, one or more modes can be combined.

### 4.3. Data Processing

Based on the sensing information, distinguishing the abnormity caused by structural and nonstructural damage is a hot topic in this field of research. Due to the sensor deviation, noise, system failures, and other coupling factors, abnormal sensing information caused by nonstructural damage must be obtained so as to provide effective information for structural damage identification and safety evaluation.

In engineering applications, as for the processing of sensor information, the methods are aimed at sensing signals. Specifically, the time domain, frequency domain, and time-frequency domain method can be adopted for noise reduction and filtering the gross error and accidental error. Also, the distorted information can be reconstructed by the trend curve method or the neural network method. Then, the missing information can be repaired by way of interpolation, substitution, and weight adjustment [27].

### 4.4. Data Management

The management of sensing information is carried out on a server which requires a fast display, efficient storage, report generation, and other functions. With an effective connection between information sensing devices and the Internet, system technologies regarding the Internet of Things can be adopted for management.

This practice has proven that a modular structure in the form of the database can achieve the hierarchical and classified management of sensing information. This database mainly includes static and dynamic databases.

(1) The static database includes information on design and construction, daily management and maintenance, system software, and hardware.
(2) The dynamic database includes information on monitoring, analysis results, and maintenance recommendations.

## 5. Bridge Safety Evaluation Based on Sensing Information

Based on the response information, theoretical calculation analysis, mathematics, and mechanics, the safety evaluation of structures can be achieved via the changing characteristics indexes of the structure itself, its response, and trends. Moreover, the individual control index can be used for a comprehensive evaluation. Some advanced methods of safety evaluation are briefly introduced as follows.

(1) Safety Evaluation Based on Reliability Theory

The structural function *Z* is constructed and the sensing information is used to modify the structural load effect model *S* and resistance model *R*. Via the reasonable statistical analysis method, the probability of exceeding the limit state during the period of usage can be obtained, which can implement the structural safety evaluation.

When *Z* = *R* – *S* > 0, it indicates that the structure is in a state of reliability.

When *Z* = *R* – *S* < 0, it indicates that the structure has been deactivated or destroyed.

When *Z* = *R* – *S* = 0, it indicates that the structure is in a limited state.

(2) Safety Evaluation Based on the Deterioration Effect

According to numerous historical monitoring data, the random process *Z_i_* is constructed, searching for and extracting the characteristic information which reflects the structural state. Then, the deterioration law of the structural performance is determined. Therefore, the following Equation (1) is used to implement the structural safety evaluation.

*Z_i_* = *μ* + *ξ_i_*, (1)

where: *μ* indicates the process mean value and *ξ_i_* indicates randomly varied parameters.

**Figure 1:**
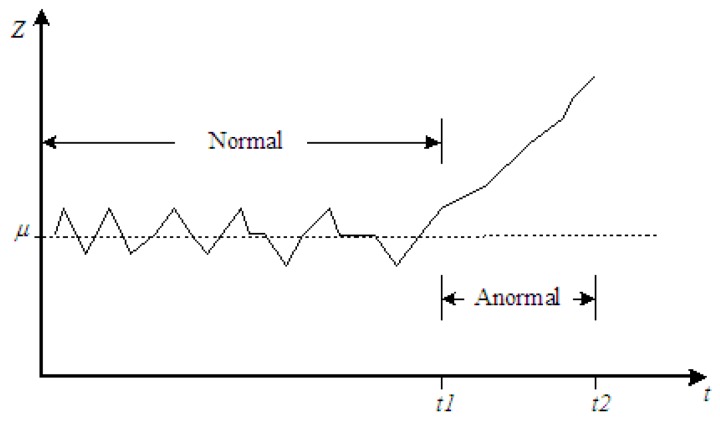
Schematic Diagram of the State of the Bridge Structure.

In Figure 1, when the structure is in a normal state, *μ* will be unchanged and *ξ_i_* will randomly vary with no obvious trend through the whole process. When damage or safety problems occur, the changing amplitude of *Z_i_* will continue to increase and *μ* will sustain an irreversible monotonic trend.

(3) Safety Evaluation Based on Envelope Theory

According to the limit state principle of bearing capacity based on the plastic theory, the design value of the unfavorable combination of the loading effect must be less than or equal to that of the structural resistance. It can be expressed as Equation (2).

(2) $$\gamma_{0}S\leq R(f_{d},\xi_{c}a_{dc},\xi_{s}a_{ds})Z_{1}(1-\xi_{e}),$$

where: $\gamma_{0}$ indicates the importance factor of the structure, *S* indicates the loading effect function, *R* indicates the resistance effect function, *f_d_* indicates the design value of material strength, *a_dc_* indicates the geometric parameter of concrete, *a_ds_* indicates the geometric parameter of rebar, *Z*_1_ indicates the calculating coefficient of the bearing capacity, *ξ_e_* indicates the deterioration coefficient of the bearing capacity, *ξ_c_* indicates the cross section reduction coefficient of the reinforced concrete structure, and *ξ_s_* indicates the cross section reduction coefficient of rebar [28].

Therefore, the loading effect diagram of each section reflected by the sensing information can be compared and analyzed with the modified limit resistance effect diagram of the bridge. The purpose is to obtain the structural safety evaluation.

(4) Safety Evaluation Based on Dynamic Response

According to the sensing information, the dynamic characteristics parameters [29] of the structure can be obtained as well as the judgment, localization, and quantification [30,31] of the structural damage. Then the finite element model can be corrected via the reanalysis of the ultimate bearing capacity, implementing the structural safety evaluation.

Regarding the fatigue damage evaluation for a steel box girder based on sensing information, the S-N curve (fatigue curve) of the construction details can be adopted to evaluate the fatigue status according to the Miner principle. Under special meteorological conditions (such as the wind, rainfall, etc.) regarding natural factors which will affect the normal service of the bridge, it is necessary to carry out this special evaluation.

Based on the sensing information, although bridge safety evaluation has obtained some great achievements which have been successfully applied to some long-span bridges, most of them focus on the overall evaluation of historical data accumulation and accidents. So we are confronted with a technical bottleneck [32] for early damage warning and online safety evaluation.

## 6. Application Analysis

### 6.1. Engineering Outline

**Figure 2:**
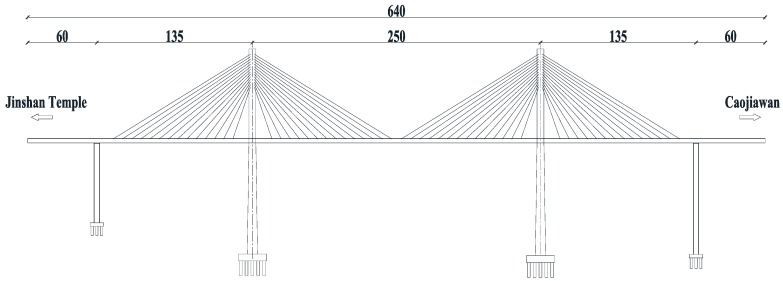
The Layout of the Main Bridge (unit: m).

The Caijia Jialing River Bridge is located in the Liangjiang New Area of the Chongqing Municipality in China. It is designed in the Rail Transit Line 6 (second-phase project) between the Jinshan Temple Station and Caojiawan Station. The total length of the bridge is 1250 m, and the main structure is a cable-stayed concrete bridge with twin towers and double cable planes where tower-beam consolidation is adopted. The layout of the spans is 60 m + 135 m + 250 m + 135 m + 60 m = 640 m, and the transverse layout is 1.5 m (cable area) + 1.4 m (maintaining road) + 4.6 m (carriageway) + 4.6 m (carriageway) + 1.4 m (maintaining road) + 1.5 m (cable area). The shape of the tower is a diamond, and the auxiliary piers are rectangular cross-sectional hollow piers. Cables adopt the steel strand and the standard strength *f_pk_* = 1860 MPa, which are protected by an HDPE (High Density Polyethylene) tube. The single cell box girder with a constant height is adopted as the main girder, and the concrete grade is C55. The layout of the main bridge is shown in Figure 2.

### 6.2. Layout and Installation of Measuring Points and Sensors

**Figure 3:**
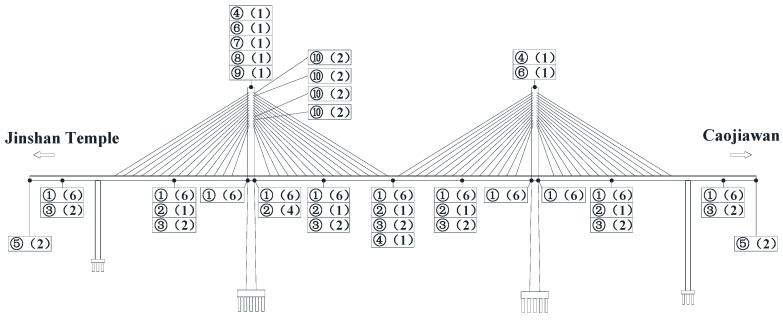
The Overall Layout of the Sensor Measuring Point. ① the stress sensor which can also measure the temperature of the structure ② the acceleration sensor ③ the static level gauge ④ GNSS (Global Navigation Satellite System) ⑤ the displacement meter ⑥ the tiltmeter ⑦ the temperature and humidity sensor ⑧ the pluviometer ⑨ the dogvane and anemoscope ⑩ the anchor cable meter through the cable. Also, the numbers in parentheses indicate the number of each sensor.

According to the principle and method of selecting controlled sections and the layout optimization of the measured points, the overall layout of the sensors in the Caijia Jialing River Bridge is shown in Figure 3.

Combined with the construction progress of the bridge, the health monitoring sensors were appropriately installed at the corresponding positions, as follows.

**Figure 4:**
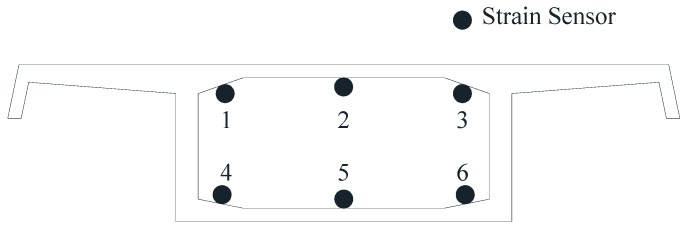
The Layout Diagram of Strain Sensors on the Surface of Concrete.

(1) On the main girder. Along the longitudinal direction of the bridge, the vibrating wire strain sensors were attached to the lower edge of the top slab and the upper edge of the bottom slab, which are shown in Figure 4. It is worth mentioned that the sensor could also measure the temperature of the structure and modify the initial temperature [33]. In addition, the correlation function [34] of the stress-time relation curve could be established by monitoring the corresponding embedded sensors in the construction process. The purpose was to obtain stress monitoring of the bridge structure under the dead load and the live load. Also, static level gauges based on the pipe principle = were installed to monitor the static long-term deformation of the girder. Meanwhile, the GNSS (Global Navigation Satellite System) was installed to monitor the spatial mid-span deformation. Also, the displacement sensors and the acceleration sensors were installed to measure the width of the expansion joint and to test the dynamic characteristics, respectively.

(2) On the cables. Before the cable tension in the construction stage and anchorage of the cable and tower, intelligent anchor cable meters with temperature measuring functions were embedded to monitor the cable force. To accurately measure the cable force under the eccentric load, the intelligent six-string type meters should be adopted.
(3) On the main tower. The tiltmeters and GNSS were installed on the top of the tower. Also, a monitoring station aimed at meteorological factors was set up. The monitoring contents included the wind speed, wind direction, rainfall, and humidity.

### 6.3. Safety Evaluation System Based on Envelope Theory

The health monitoring for the Caijia Jialing River Bridge considered loading factors including live train load, transverse rocking force, braking force, temperature force, wind load, water pressure [35,36], etc. Via the finite element simulation analysis by the professional program Midas/Civil, the sub loads were combined to establish the upper and lower bound of the envelope interval. For example, for the main girder of the bridge, the vertical deformation and the longitudinal stress of point 2 (middle position of the top slab) and point 5 (middle position of the bottom slab) were measured.

**Figure 5:**
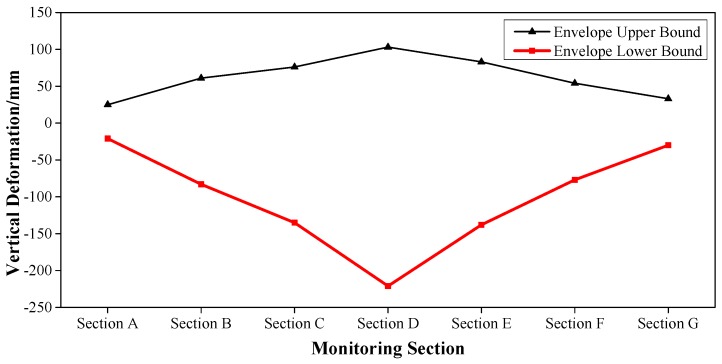
Envelope Upper and Lower Bounds of the Vertical Deformation for the Main Girder.

**Figure 6:**
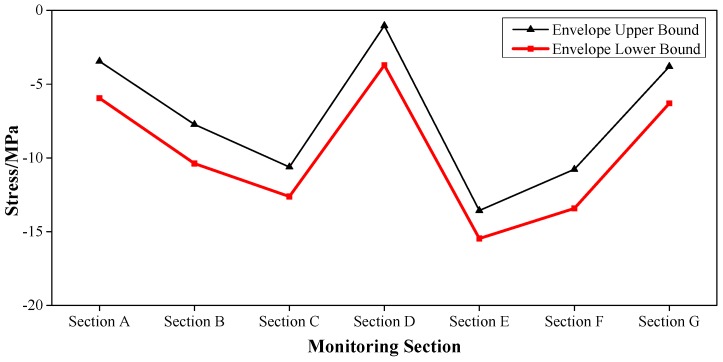
Envelope Upper and Lower Bounds of the Stress of Point 2 for the Main Girder.

**Figure 7:**
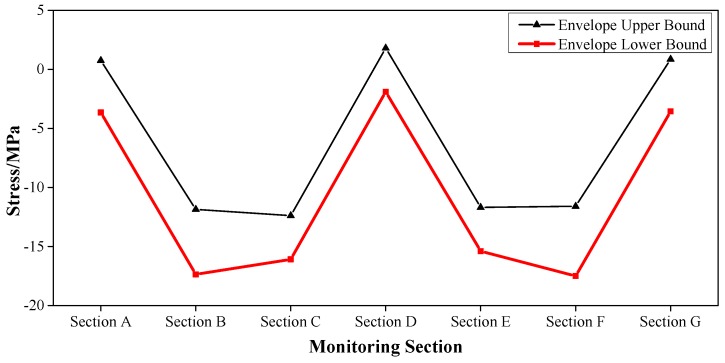
Envelope Upper and Lower Bounds of the Stress of Point 5 for the Main Girder.

These results are shown in Figure 5, Figure 6 and Figure 7, where Section A–Section G successively represent: L/2 Secondary Sidespan next to Jinshan Temple, L/2 Sidespan next to Jinshan Temple, L/4 Mid-span, L/2 Mid-span, 3L/4 Mid-span, L/2 Sidespan next to Caojiawan, and L/2 Secondary Sidespan next to Caojiawan.

Based on the upper and lower bounds of the envelope interval, the safety coefficient *r* = 0.75, was determined. Then the envelope intervals [37] of a normal state, critical state, and degradation evaluation were established. For instance, for the vertical stiffness of the mid-span, the calculation results for the deformation in the Z-direction (DZ) are shown in Table 2.

So in the mid-span of the main girder, the envelope of the vertical stiffness was [−166 mm, 77 mm] in the normal state and was [−221 mm, −166 mm) ∪ (77 mm, 103 mm] in the critical state.

Similarly, the envelope intervals of other physical parameters such as the stress and cable forces could be established. The time-varying effect among the material characteristics was considered. With the continuous operation time, the results of the daily inspection were also organically integrated. Then, the coefficient of checking and calculation, the deterioration coefficient of the bearing capacity, the reduction coefficient of the sections, and the influence coefficient of the train live load [28] were introduced and the envelope interval could be reasonably revised.

**Figure 8:**
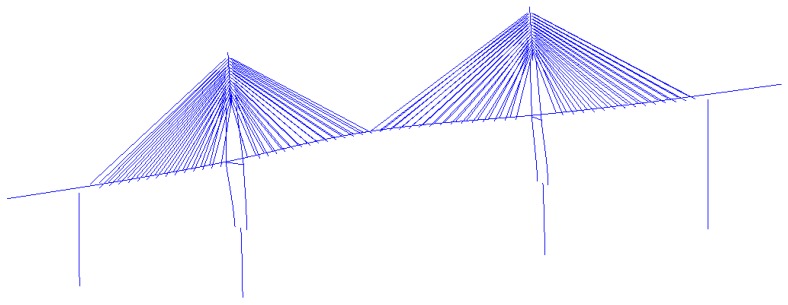
The First-order Vibration Mode.

Meanwhile, based on the finite element model, the static and dynamic characteristics were analyzed. The natural vibration characteristics were taken as an example [39]. The first-order vibration mode is shown in Figure 8, and the first five orders of the frequency and vibration mode are shown in Table 3.

### 6.4. Monitoring and Evaluation

When installed completely, the health monitoring system should be debugged as a whole. Before the passage of the train, the time 02:00 in the early morning would be regarded as the reference time, which would commence the acquisition of monitoring information constantly, online, and dynamically. For example, the GNSS automatically collected data at 10 s intervals. Meanwhile, the acceleration sensors automatically collected data continuously. Other monitoring contents including the stress and the cable force were collected at 10 min intervals. Then, the monitoring information could be transmitted to the server by way of wired transmission. Ultimately, the processing and evaluation of data was implemented.

One day is taken as an example. The monitoring and evaluation of some measured parameters are explained.

**Figure 9:**
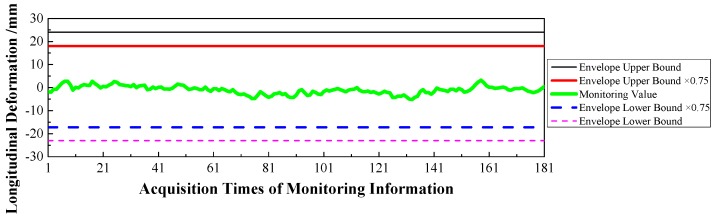
Longitudinal Deformation of the Mid-span.

**Figure 10:**
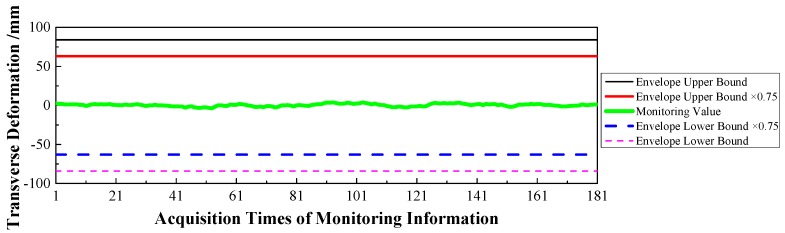
Transverse Deformation of the Mid-span.

**Figure 11:**
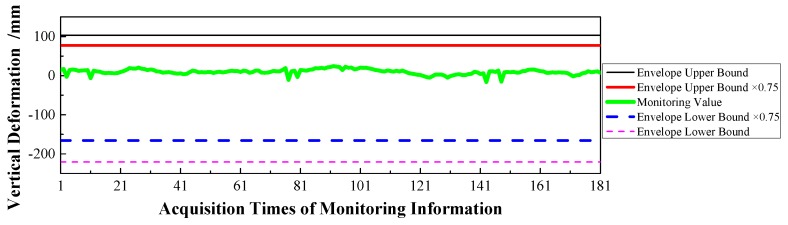
Vertical Deformation of the Mid-span.

**Figure 12:**
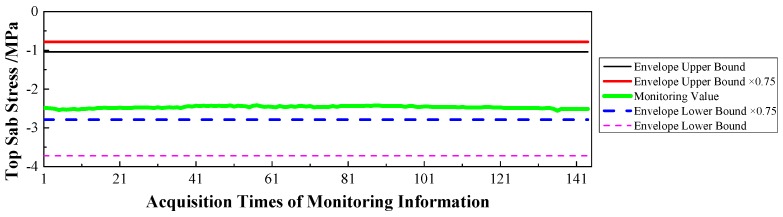
Top Slab Stress of the Mid-span.

**Figure 13:**
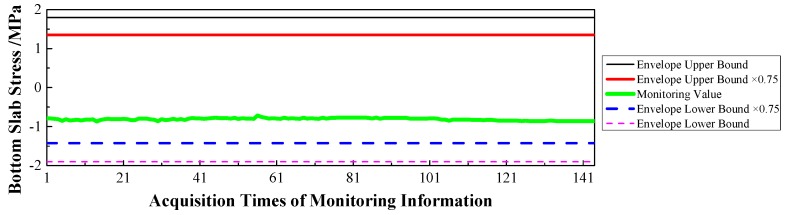
Bottom Slab Stress of the Mid-span.

**Figure 14:**
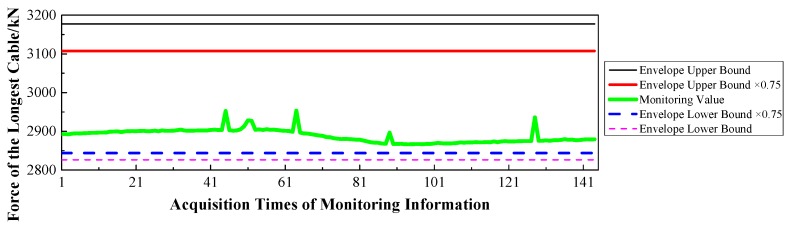
Force of the Longest Cable.

**Figure 15:**
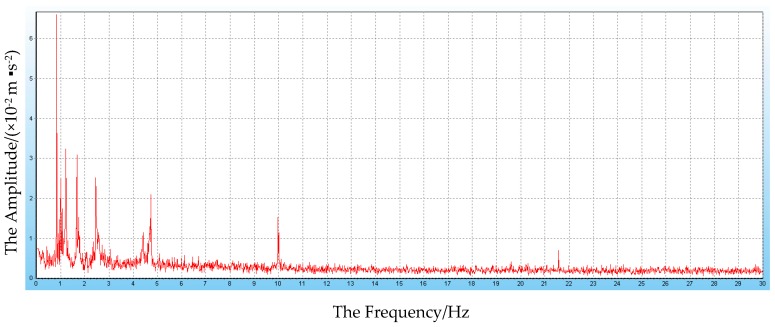
Dynamic Characteristics.

(1) Displacement: via the baseline calculation software (spider) and analysis software (GeoMoS), the spatial deformation information of the mid-span collected by GNSS could be changed into the longitudinal, transverse, and vertical deformation with respect to the reference time. After the correction of the temperature factor, the monitoring information would enter the evaluation system, and the results are shown in Figure 9, Figure 10 and Figure 11.
(2) Stress: the strain information would be collected by the vibrating wire strain sensor (including the function of temperature). Then, according to the constitutive relationship between stress and strain, the stress of the structure would be calculated under the loads. Via the evaluation system, the evaluation results of the top slab and bottom slab stress along the longitudinal direction in the mid-span are shown in Figure 12 and Figure 13.
(3) Cable force: the measured information from the embedded and intelligent anchor cable meter would be corrected by the temperature, and the real cable force would be obtained. Then via the evaluation system, the monitoring and evaluation results of the longest cable are shown in Figure 14.
(4) Dynamic characteristics: when the temperature was stable and no train was passing, the acceleration signal was sampled. Then, the signal was transformed by FFT (Fast Fourier Transform), and the natural frequency of vibration was obtained. The monitoring result is shown in Figure 15.

In order to clearly describe the results of the static characteristics, the evaluation coefficient F is defined in Equation (3).

(3) $$F=\frac{M}{N},$$

where: M indicates the measured value of the loading effects, N indicates the theoretical value of the loading effects. When F is less than or equal to 1, it indicates that the working performance of the structure is good. When F is greater than 1, it indicates that the working performance is not ideal and some defects may exist.

Therefore, based on Figure 9, Figure 10, Figure 11, Figure 12, Figure 13, Figure 14 and Figure 15 and combined with the evaluation coefficient F, the conclusions can be drawn as follows.

(1) Based on Figure 9, which was aimed at longitudinal deformation in the mid-span section, the measured extreme $M_{\max1}$ was −5 mm which meant the bridge was deviating to the river bank, and it was located in the normal state envelope interval [−17 mm, 17 mm]. The theoretical extreme $N_{\max1}$ was −17 mm, so $F_{1}$ was less than 1, which indicated that the longitudinal stiffness was normal.
(2) Based on Figure 10 which was aimed at the transverse deformation in the mid-span section, the measured extreme $M_{\max2}$ was 4 mm which meant the bridge was deviating from upstream to downstream, and it was located in the normal state envelope interval [−63 mm, 63 mm]. The theoretical extreme $N_{\max2}$ was 63 mm, so $F_{2}$ was less than 1, which indicated that the transverse stiffness was normal.
(3) Based on Figure 11 which was aimed at the vertical deformation in the mid-span section, the measured extreme $M_{\max3}$ was −24 mm which meant the bridge was deviating from upstream to downstream, and it was located in the normal state envelope interval [−166 mm, 77 mm]. The theoretical extreme $N_{\max3}$ was −166 mm, so $F_{3}$ was less than 1, which indicated that the vertical stiffness was normal.
(4) Based on Figure 12 which was aimed at the top slab stress (point 2) in the mid-span section, the measured extreme $M_{\max4}$ was −2.56 MPa which indicated compressive stress, and it was located in the normal state envelope interval [−2.79 MPa, −0.78 MPa]. The theoretical extreme $N_{\max4}$ was −2.79 MPa, so $F_{4}$ was less than 1, which indicated that the stiffness of the top slab was normal.
(5) Based on Figure 13 which was aimed at the bottom slab stress (point 5) in the mid-span section, the measured extreme $M_{\max5}$ was −0.87 MPa which indicated compressive stress, and it was located in the normal state envelope interval [−1.43 MPa, 1.35 MPa]. The theoretical extreme $N_{\max5}$ was −1.43 MPa, so $F_{5}$ was less than 1, which indicated that the stiffness of the bottom slab was normal.
(6) Based on Figure 14 which was aimed at the cable force of the longest cable, the measured extreme $M_{\max6}$ was 2953.6 kN and it was located in the normal state envelope interval [2843.9 kN, 3107.3 kN]. The theoretical extreme $N_{\max6}$ was 3107.3 kN, so $F_{6}$ was less than 1, which indicated that the cable force was normal.

In a word, when combined with the results above, the evaluation coefficient $F=\{F_{1},F_{2},F_{3},F_{4},F_{5},F_{6}\}$ was less than 1. So the static characteristics of the bridge were normal.

Meanwhile, based on Figure 15, the measured first mode frequency (0.835 Hz) was greater than the calculated frequency (0.686 Hz). It indicated that the dynamic characteristics were normal.

In summary, the static and dynamic characteristics of the Caijia Jialing River Bridge were all normal. The bridge structure was also in a normal state.

## 7. Conclusions

(1) The rapid development of sensor technology has provided strong technical support for the health monitoring and evaluation of long-span bridges. It is urgent to adopt new theories and new effects so as to study and develop bridge monitoring sensing devices which are reliable, stable, and durable.
(2) The health monitoring and evaluation projects of long-span bridges have been spawned by many committed companies. They are immersed in this smooth and prosperous situation. Moreover, the monitoring system including the sensors and the collection, transmission, processing, and management of sensing information, also has its own unique characteristics, advantages, and disadvantages, which are difficult to evaluate effectively. So following the maximization principle of the “cost-benefit ratio”, the optimization of the design can be carried out, and the monitoring standardization system can be established to realize standardization.
(3) The analysis of the engineering application demonstrates that the comprehensive evaluation can be effectively combined with individual control indicators. Also, the objective evaluation of the bridge state can be conducted. However, for long-span bridges, using new ideas and concepts to achieve practical safety evaluation techniques based on massive sensor information are still great challenges for engineers to overcome, including conducting structural damage prognosis and safety prognosis studies [40].

## Figures and Tables

**Table 1:** The Health Monitoring Contents of Long-Span Bridges.

		Bridge Type	Girder Bridge	Arch Bridge	Cable-Stayed Bridge	Suspension Bridge
Categories						
load and environment	moving load		★	★	★	★
	earthquake		★	★	★	★
	impact		★	★	★	★
	temperature		▲	▲	▲	▲
	humidity		▲	▲	▲	▲
	wind speed and direction		◇	★	▲	▲
	rainfall		★	★	★	★
the overall response of the structure	vibration		▲	▲	▲	▲
	deformation		▲	▲	▲	▲
	rotation angle		◇	◇	★	★
the local response of the structure	stress		▲	▲	▲	▲
	cable force		○	▲	▲	▲
	crack		★	★	★	★
	fatigue		○	★	★	★
	corrosion		★	★	★	★
	changing and the reaction of supports		★	★	★	★
	foundation erosion		★	★	★	★

Illustrating: ▲ indicates the item which shall be monitored. ★ indicates the item which is selected to be monitored if the condition permits. ◇ indicates the item which can be monitored. ○ indicates the item which is unnecessary to be monitored.

**Table 2:** Deformation in the Z-direction (DZ) for Mid-span of the Main Girder (unit: mm).

Load		DZ	*r*DZ
train live load	min	−154	−115
	max	31	23
transverse rocking force	+	0	0
	−	0	0
braking force	+	0	0
	−	0	0
temperature load [38]	system temperature rising	21	16
	system temperature dropping	−21	−16
	component temperature rising	−41	−31
	component temperature dropping	41	31
	gradient temperature rising	10	7
	gradient temperature dropping	−5	−4
wind load	transverse wind +	0	0
	transverse wind −	0	0
	longitudinal wind +	0	0
	longitudinal wind −	0	0
	train wind +	0	0
	train wind −	0	0
water pressure	+	0	0
	−	0	0

Illustrating: down-warping is negative, otherwise, positive.

**Table 3:** Analysis of Natural Vibration Characteristics of the Bridge.

Order	Calculated Frequency (Hz)	Period (s)	Description of Vibration Mode
1	0.686	1.457	Transverse Vibration of Bridge Tower + Transverse Bending of Beam
2	0.752	1.330	Anti-symmetric Transverse Vibration of Bridge Tower + Transverse Bending of Beam
3	0.8702	1.149	Longitudinal Drift of the Beam
4	0.8852	1.130	First-order Symmetric Vertical Bending
5	1.1352	0.881	First-order Symmetric Transverse Bending

## References

[1] Li H., Zhou W.S., Ou J.P., Yang Y. (2006). A study on system integration technique of intelligent monitoring systems for soundness of long-span bridges. China Civ. Eng. J..

[2] Li Z.M., Li J.X., Chen R.Z. (2008). Research of Bridge Structural Health Monitoring Data Acquisition System Design Method. Sci. Technol. Eng..

[3] Chen B., Wang X., Sun D., Xie X. (2014). An integrated system for structural health monitoring and intelligent management for a cable-stayed bridge. Sci. World J..

[4] Hui L.I., Ou J. (2011). Structural health monitoring: From sensing technology stepping to health diagnosis. Procedia Eng..

[5] Ko J.M., Ni Y.Q. (2005). Technology developments in structural health monitoring of large-scale bridges. Eng. Struct..

[6] Kashima S., Yanaka Y., Suzuki S. (2001). Monitoring the Akashi Kaikyo Bridge: First Experiences. Struct. Eng. Int..

[7] Zhao X., Liu H., Yu Y., Xu X., Hu W., Li M., Ou J. (2015). Bridge displacement monitoring method based on laser projection-sensing technology. Sensors.

[8] Ma H.W., Nie Z.H. (2015). Research progress and reflection on safety monitoring of bridges. Mech. Eng..

[9] Zhang X. (2014). Health monitoring of bridge structure. Traffic World..

[10] Li H., Ren L., Jia Z., Yi T. (2016). State-of-the-art in structural health monitoring of large and complex civil infrastructures. J. Civ. Struct. Health Monit..

[11] Li P.F., Wu T.C. (2011). Review of bridge health monitoring technology. Prestress. Technol..

[12] Mufti A. (2001). Guidelines for Structural Health Monitoring.

[13] Aktan A.E., Catbas F.N., Grimmelsman K.A. (2012). Development of a Model Health Monitoring Guide for Major Bridges.

[14] Rucker W., Hille F., Rohrmann R. (2006). Guideline for Structural Health Monitoring.

[15] Ministry of Transport of the People’s Republic of China (2016). Technical Specification for Structural Safety Monitoring Systems of Highway Bridges (JT/T 1037-2016).

[16] Li J., Deng J., Xie W. (2015). Damage Detection with Streamlined Structural Health Monitoring Data. Sensors.

[17] Sun X.Y. (2006). Research Progress on health monitoring technology of bridge structure. J. China Foreign Highw..

[18] Wang H., Tao T.Y., Li A.Q. (2016). Structural health monitoring systems for Sutong Cable-stayed Bridge. Smart Struct. Syst..

[19] Li J., Hao H., Fan K.Q. (2014). Development and application of a relative displacement sensor for structural health monitoring of composite bridges. Struct. Control Health Monit..

[20] Lu Z.Z., Zhou J.T., Chen Y. (2015). Dynamic Stress Monitoring Sensor of Multi-direction Concrete. Chinese Patent.

[21] Guyan R.J. (1995). Reduction of stiffness and mass matrices. AIAA J..

[22] Breitfeld T. (1996). A method for identification of a set of optimal measurement points for experimental modal analysis: Modal analysis. Int. J. Anal. Exp. Modal Anal..

[23] Johnson G.W., Jennings R. (2002). LabVIEW Graphics Programming.

[24] National Instruments Corporation (1998). Labwindows/CVI Basics I.

[25] Araujo A., Garcia-Palacios J., Blesa J. (2012). Wireless Measurement System for Structural Health Monitoring With High Time-Synchronization Accuracy. IEEE Trans. Instrum. Meas..

[26] Whelan M.J., Gangone M.V., Janoyan K.D. (2009). Highway Bridge Assessment Using an Adaptive Real-Time Wireless Sensor Network. IEEE Sens. J..

[27] Zhou J., Zhang J., Liu S. (2014). Theory and Technology of Practical Monitoring and Evaluation for Large Bridge.

[28] Ministry of Transport of the People’s Republic of China (2011). Specification for Inspection and Evaluation of Load-Bearing Capacity of Highway Bridges (JTG/T J21-2011).

[29] Yang J., Zhou Y., Zhou J., Chen Y. (2013). Prediction of Bridge Monitoring Information Chaotic Using Time Series Theory by Multi-step BP and RBF Neural Networks. Intell. Autom. Soft Comput..

[30] Khorram A., Bakhtiari-Nejad F., Rezaeian M. (2012). Comparison studies between two wavelets based crack detection methods of a beam subjected to a moving load. Int. J. Eng. Sci..

[31] Rao M.A., Srinivas J., Murthy B.S. (2004). Damage detection in vibrating bodies using genetic algorithms. Comput. Struct..

[32] Ou J.P., Li H. (2010). Structural health monitoring in mainland China: Review and future trends. Struct. Health Monit..

[33] Yang J., Zhang H., Zhou J.T. (2016). Discussing the Initial Temperature Difference Correction Method for Vibrational Chord Strain Gauge in Bridge Construction Monitoring. Intell. Autom. Soft Comput..

[34] Yang J., Zhang H., Zhou J., Xu B., Shan A. (2016). Discussing the initial temperature difference correction method for vibrational chord strain gauge in bridge construction monitoring. Intell. Autom. Soft Comput..

[35] Ministry of Railways of the People’s Republic of China (2005). Code for Design on Reinforce and Prestressed Concrete Structure of Railway Bridge And Culvert (TB10002.3-2005).

[36] Ministry of Railways of the People’s Republic of China (2005). Fundamental Code for Design on Railway Bridge and Culvert (TB10002.1-2005).

[37] Li X.G., Hui D., Zhou J.T. (2016). Construction and Application of the Health Monitoring Evaluation System of Long-Span Cable-Stayed Bridge. J. China Foreign Highw..

[38] Xiang H. (2001). Advanced Bridge Structural Theory.

[39] Li X.G., Zhou J.T., Yang J., Xiao Y.J. (2016). Analysis of Static and Live Load Pre-camber Setting for Long-span Railway Cable-stayed Bridge Based on the Dynamic Characteristics. Sci. Technol. Eng..

[40] Zong Z.H., Zhong R.M., Zheng P.J. (2014). Damage and Safety Prognosis of Bridge Structures Based on Structural Health Monitoring: Progress and Challenge. J. China Foreign Highw..

